# A randomized, controlled trial of spinal endoscopic adhesiolysis in chronic refractory low back and lower extremity pain [ISRCTN 16558617]

**DOI:** 10.1186/1471-2253-5-10

**Published:** 2005-07-06

**Authors:** Laxmaiah Manchikanti, Mark V Boswell, Jose J Rivera, Vidya Sagar Pampati, Kim S Damron, Carla D McManus, Doris E Brandon, Sue R Wilson

**Affiliations:** 1 Pain Management Center of Paducah, 2831 Lone Oak Road, Paducah, Kentucky, 42003, USA; 2 Case University School of Medicine, University Hospitals of Cleveland, 11100 Euclid Avenue, Cleveland, OH, 44106, USA

## Abstract

**Background:**

Postoperative epidural fibrosis may contribute to between 5% to 60% of the poor surgical outcomes following decompressive surgery. Correlations have been reported between epidural scarring and radicular pain, poor surgical outcomes, and a lack of any form of surgical treatment. The use of spinal endoscopic adhesiolysis in recent years in the management of chronic refractory low back and lower extremity pain has been described.

**Methods:**

A prospective, randomized, double-blind trial was conducted to determine the outcome of spinal endoscopic adhesiolysis to reduce pain and improve function and psychological status in patients with chronic refractory low back and lower extremity pain. A total of 83 patients were evaluated, with 33 patients in Group I and 50 patients in Group II. Group I served as the control, with endoscopy into the sacral level without adhesiolysis, followed by injection of local anesthetic and steroid. Group II received spinal endoscopic adhesiolysis, followed by injection of local anesthetic and steroid.

**Results:**

Among the 50 patients in the treatment group receiving spinal endoscopic adhesiolysis, significant improvement without adverse effects was shown in 80% at 3 months, 56% at 6 months, and 48% at 12 months. The control group showed improvement in 33% of the patients at one month and none thereafter. Based on the definition that less than 6 months of relief is considered short-term and longer than 6 months of relief is considered long-term, a significant number of patients obtained long-term relief with improvement in pain, functional status, and psychological status.

**Conclusion:**

Spinal endoscopic adhesiolysis with targeted delivery of local anesthetic and steroid is an effective treatment in a significant number of patients with chronic low back and lower extremity pain without major adverse effects.

## Background

Postoperative epidural fibrosis, the formation of dense scar tissue adjacent to the dura mater following surgical laminectomy, may play a role in up to 60% of the poor surgical outcomes following decompressive surgery [1-3]. A correlation between peridural scarring and radicular pain [4-7], and poor clinical outcomes [8,9] has been reported by some, while others [10-13] have questioned the role of epidural fibrosis as a causative factor. Increased complication rates have been reported with revision spine surgery with increased occurrence of dural tears, nerve root injury, and bleeding [14,15]. Phillips and Cunningham [16] reported that no form of surgical treatment or adhesion lysis procedure was safe or effective for post-lumbar laminectomy syndrome.

Epidural fibrosis results from the invasion of postoperative hematoma by dense fibrous tissue originating from the periosteum and within the deep surface of the paravertebral musculature [17,18]. Epidural fibrosis may extend into the neural canal and adhere to the dura mater and nerve roots, with mechanical tethering of nerve roots or dura by adhesions, which may in turn contribute to persistent back and leg pain following lumbar laminectomy. However, epidural fibrosis also may develop without surgical intervention, secondary to annular tear, hematoma, infection, or intrathecal contrast media [18-20]. Perineural fibrosis can render nerve roots hyperesthetic and hypersensitive to compression forces by interfering with cerebrospinal fluid-mediated nutrition [6] or by making the nerve susceptible to injury [7].

A moderate proportion of patients show improvement in pain and functional level with interventional pain management procedures, including fluoroscopically-directed epidural steroid injections and percutaneous adhesiolysis utilizing a special catheter [21,22]. In addition, initial clinical studies of spinal endoscopic adhesiolysis [23-29] and a preliminary report of a randomized controlled trial [30] showed improved clinical outcomes. However, a recent prospective, randomized, double-blind trial [31] comparing caudal epidural with targeted steroid placement on affected nerve roots during spinal endoscopy for chronic sciatica, concluded that targeted placement of steroid did not significantly reduce pain intensity and anxiety and depression compared with caudal epidural steroid injection.

Most studies utilized post lumbar laminectomy syndrome or epidural fibrosis as inclusion criteria, whereas one study [28] included patients with lumbar spinal stenosis, and another [31] exclusively studied patients without history of surgery. In addition, some studies [26,27,30] specifically described inclusion criteria as patients without long-term improvement following fluoroscopically directed epidural steroid injections and one-day percutaneous adhesiolysis. Consequently, these studies represent heterogenous populations. Even though retrospective evaluations [26,27] have shown the effectiveness of spinal endoscopic adhesiolysis in patients after lack of long-term effect following fluoroscopically-directed caudal epidural steroid injections and one-day percutaneous adhesiolysis, the effectiveness has not been demonstrated in controlled trials.

Spinal endoscopy also has been utilized for diagnostic purposes. Even though multiple authors have described various types of findings, including the identification of inflammation with an endoscope, neither the reliability nor the clinical utility of spinal endoscope as a diagnostic tool has been established [23-25,28,29]. Consequently, no attempt was made to evaluate the diagnostic utility of spinal endoscopy.

This randomized, double-blind, controlled trial of spinal endoscopic adhesiolysis and targeted delivery of steroids was designed to evaluate their effectiveness in patients with chronic low back and lower extremity pain who lacked significant response to fluoroscopically-directed epidural steroid injections and one-day percutaneous adhesiolysis with hypertonic saline neurolysis, as well as to other conservative modalities of treatment.

## Methods

This study was designed to evaluate the effectiveness of spinal endoscopic adhesiolysis in chronic, refractory low back and lower extremity pain. The study was undertaken in an interventional pain management practice (a specialty referral center) in a private practice setting, in accordance with the guidelines for randomized controlled trials [32,33], and the quality checklists of systematic reviews [32,34-39]. The trial also was designed to meet criteria of a pragmatic or a practical clinical trial [30,40,41]. The protocol was approved by the Institutional Review Board of the Ambulatory Surgery Center where the study was conducted. The objective was to evaluate the effectiveness of spinal endoscopic adhesiolysis compared to caudal epidural steroid injection. The design consisted of a control group and an intervention group. Group I (control group) was treated with introduction of the spinal endoscope up to the S3 level, followed by injection of a local anesthetic and steroid. Group II (intervention group) was treated with appropriate spinal endoscopic adhesiolysis at L4, L5 or S1 level(s) unilaterally or bilaterally based on symptomatology, followed by a targeted injection of local anesthetic and steroid.

### Inclusion and exclusion criteria

The majority of participants in this study were identified from existing patients of the interventional pain management practice. Eligible new patients were screened and identified as candidates for the program.

#### Inclusion criteria

patients between 18 and 65 years of age with a history of chronic low back and lower extremity pain of at least two year's duration, without facet joint pain based on controlled comparative local anesthetic blocks, and without significant improvement with conservative treatment including fluoroscopically-directed epidural injections and one-day percutaneous adhesiolysis with hypertonic saline neurolysis, and willingness to participate in the clinical trial were enrolled.

Criteria for lack of significant response to caudal epidural steroid injections was pain relief (≥ 50%) for one week or less following a second epidural steroid injection, and relief of four weeks or less following a third epidural steroid injection or any of subsequent epidural steroid injections. Criteria for lack of response to one-day percutaneous adhesiolysis with hypertonic saline neurolysis was considered as lack of response to the first adhesiolysis procedure, less than two months of pain relief (≥ 50%) following the second or subsequent procedures.

#### Exclusion criteria

patients with cauda equina syndrome, compressive radiculopathy, surgical intervention in the previous six months, opioid abuse and dependency evaluated by adherence monitoring, including random drug testing and opioid use of no greater than hydrocodone 100 mg per day, methadone 60 mg or morphine 100 mg or equivalent doses of other drugs; uncontrolled major depression or psychiatric disorders; uncontrolled or acute medical illnesses including severe cardiac, pulmonary, or other disorders; chronic conditions that could interfere with the interpretations of the outcome assessments such as severe hip or knee arthritis, neuropathy, or other disorders; pregnant or lactating women; history of adverse reaction to local anesthetic or steroids; inability to understand the informed consent and protocol; or inability to be positioned in the prone position during the procedure [30].

### Evaluation

All patients were provided with the protocol and the informed consent document approved by the Institutional Review Board for this study. The informed consent document described the details of the trial.

The screening evaluation consisted of demographic data, medical/surgical history with co-existing diseases, radiographic investigations, physical examination, psychological evaluation with Pain Patient Profile (P-3^®^), Visual Analogue Scale (VAS) pain scores, work status, Oswestry Disability Index 2.0, and lumbar spine range of motion with ARCON ROM computerized dual inclinometer system, based on AMA "Guides to the Evaluation of Permanent Impairment" validity criterion utilizing three consecutive measurements with ± 5° or ± 10% of mean value.

### Interventions

All patients in both groups were provided identical preparation. All procedures were performed using fluoroscopy in an ambulatory surgery center in sterile operating rooms by one physician (LM).

### Procedure

The procedure included appropriate preparation with intravenous access, pre-procedure antibiotic administration, sterile preparation, and appropriate sedation with fentanyl and midazolam. Access to the epidural space was obtained with a RK^® ^needle. An epidurogram was obtained which identified filling defects and/or epidural fibrosis. Adhesiolysis was carried out in the intervention group utilizing the myeloscope^® ^spinal endoscopic video-guided catheter system and introducer system, with final positioning of the fiberoptic endoscope on the side and level of the defect and the source of pain with an additional injection of contrast to identify adhesiolysis, followed by targeted injection of local anesthetic and steroid.

Following initial epidurography in the control group, a 0.9 mm guidewire was inserted through the needle, which was advanced under fluoroscopic guidance to S3 level. Then, a 2-mm × 17.8-cm dilator with catheter (sheath) was passed over the guidewire again up to S3. At that time, a 0.8-mm fiberoptic spinal endoscopic video-guided system was introduced into the catheter through the valve and was advanced until the tip was positioned at the distal end of the catheter through the valve, as determined by video and fluoroscopic images not to exceed S3. Following this, 10 mL of 1% lidocaine and 6 mg to 12 mg of betamethasone or 40 mg to 80 mg of methylprednisolone were injected through the epiduroscope. Following the completion of the procedure, the endoscope was removed and appropriate sterile Bioclusive dressing was applied.

In the intervention group, following initial epidurography, a 0.9-mm guidewire was inserted through the needle (occasionally facilitated by a small incision with a #11 straight blade), which was advanced under fluoroscopic guidance to the level of suspected pathology. Following this, a 2-mm × 17.8-cm dilator with catheter (sheath) was passed over the guidewire. Once the catheter was advanced to the tip of the guidewire, the wire was removed. A 0.8-mm fiberoptic spinal endoscopic video-guided system was introduced into the catheter through the valve and advanced until the tip was positioned at the distal end of the catheter, as determined by video and fluoroscopic images. In conjunction with gentle irrigation using normal saline, the catheter and fiberoptic myeloscope were manipulated and rotated in multiple directions, with visualization of the nerve roots at various levels. Painful nerve root was confirmed by endoscopic manipulation, based on pre-procedural clinical and radiographic evaluation. Adhesiolysis and decompression were carried out by distension of the epidural space with normal saline and by mechanical means utilizing the fiberoptic endoscope. Adhesiolysis was confirmed by injection of non-ionic contrast material (Omnipaque 240^®^) and an epidurogram was performed on at least two occasions. The volume of sodium chloride solution utilized for irrigation was closely monitored. The protocol limited the total volume of contrast and sodium chloride solution not to exceed 100 mL. Adhesiolysis was limited to L4, L5 or S1 levels, either unilaterally or bilaterally. Following completion of the procedure, 4 mL to 8 mL of lidocaine 1%, preservative free, mixed with either 6 mg or 12 mg of betamethasone or 40 mg or 80 mg of methylprednisolone was injected after assuring that there was no evidence of subarachnoid leakage of contrast. The injection of betamethasone or methylprednisolone was based on its availability in the market. Methylprednisolone was utilized if betamethasone was not available. If pathology was identified at multiple levels, the procedure was carried out at those levels, and the injectate was given in divided doses. If there was a question of subarachnoid leakage of the contrast, a Racz catheter^® ^was passed into the epidural space, and a mixture of local anesthetic was injected very slowly in incremental doses, followed by injection of the steroid if satisfactory follow-up was obtained without any subarachnoid blockade. Following the injection of local anesthetic and steroid, the scope was removed and appropriate sterile Bioclusive dressing was applied.

### Co-interventions

No specific co-interventions were offered. Baseline drug therapy was allowed to be continued with no changes being made towards increasing opioids until after the unblinding and/or documented failure of intervention. However, opioid decreases were implemented based on improvement in functional status and reduction in pain following the interventions. Self-directed exercises as tolerated were also prescribed.

### Outcomes assessment

Outcomes were assessed at 3-month, 6-month, and 12-month intervals post-treatment with the Visual Analogue Scale (VAS) pain scale, Oswestry Disability Index 2.0, work status, opioid intake, range of motion measurement by ARCON ROM computerized evaluation, and psychological evaluation by P-3. They were compared to baseline within both groups and with each other at various time intervals. Duration of relief was judged to be short-term, if relief was less than 6 months. If relief lasted for at least 6 months, it was considered long-term. Significant relief was defined as pain relief of 50% or greater.

VAS was measured on a 10 cm scale. P-3 psychological evaluation [42] and Oswestry Disability Index 2.0 [43] were assessed by administration of appropriate questionnaires. Range of motion was evaluated by a certified physical therapist, blinded to the type of treatment. Based on P-3, scores of 55 or higher were considered positive for a diagnosis of depression, whereas, scores of 56 or higher were considered to provide the diagnosis of anxiety or somatization.

Opioid intake was determined as none, mild, moderate, or heavy based on the dosage, frequency and schedule of the drug as follows: Considered as mild was an intake of Schedule IV opioids, i.e., propoxyphene napsylate, pentazocine hydrochloride, tramadol hydrochloride up to a maximum of four times, or hydrocodone less than 40 mg per day; considered moderate was an intake of Schedule III opioids, i.e., hydrocodone, up to 40 mg per day; and considered heavy was an intake of Schedule II opioids, i.e., oxycodone, morphine, meperidine, transdermal fentanyl, and methadone, in any dosage.

Employment and work status (employed, unemployed, housewife, disabled, and retired) were determined from the pre-treatment and post-treatment work status. Only employed and unemployed patients were considered to be eligible for employment, whereas disabled patients and retired patients were considered not employable. Patients in the "housewife" category were considered neither employable nor unemployable.

## Statistical methods

### Study design

Randomization was 2:3 with two patients randomized to the control group (Group I), for every three patients randomized to the spinal endoscopic adhesiolysis group (Group II). Randomization was performed by the statistician using a computer-generated random allocation sequence in blocks of 15 patients.

The random allocation was concealed from the physician doing the procedure and personnel in the operating room until the intervention. Randomization was not revealed to the personnel in the recovery room or to the reviewing physician. After treatment, the patient was never in contact with anyone with knowledge to the randomization assignment, until after the unblinding was performed.

Unblinding was considered at a patient's request and/or treatment was considered a failure at 3 months or later. All other patients were unblinded at 12 months. Patients were also given an option to discontinue or to withdraw from the study for any and all reasons. They were considered withdrawn if follow-up was lost.

### Intent-to-treat analysis

An intent-to-treat analysis was performed by including all subjects by carrying forward the last observation.

### Statistical analysis

Demographic data were analyzed by means of the Student's t test and the chi-squared test. Fischer's exact test was used wherever the expected value was less than five. For analyzing Outcome measurement based on Visual Analogue Scale (VAS) Report and Oswestry Disability Index, range of motion (ROM), depression, anxiety and somatization scores, student's t test (parametric) and The Mann-Whitney U test (non-parametric) were used to test mean differences between groups. A paired t test and Wilcoxon signed-rank test were used to compare pre-and post-treatment results for individual patients. When results from both parametric and non-parametric tests were similar, *P *values from the parametric tests were reported in the tables and text. Results were considered statistically significant if the *P *value was less than 0.05.

## Results

The study was conducted from January 2002 through December 2003. As per the protocol, initial results were published in 2003; this preliminary report included a 6 month follow up with 16 patients in caudal epidural steroid injection group, and 23 patients in spinal endoscopy group [30]. To facilitate this publication, results of two patients in the control group and 12 patients in the intervention group were unblinded by the statistician for purposes of the evaluation and preliminary publication. This unblinding was not revealed to investigators, other staff, and the participants of the study. Consequently, 39 patients reported at 6 months were also included in the present report.

**Figure 1:**
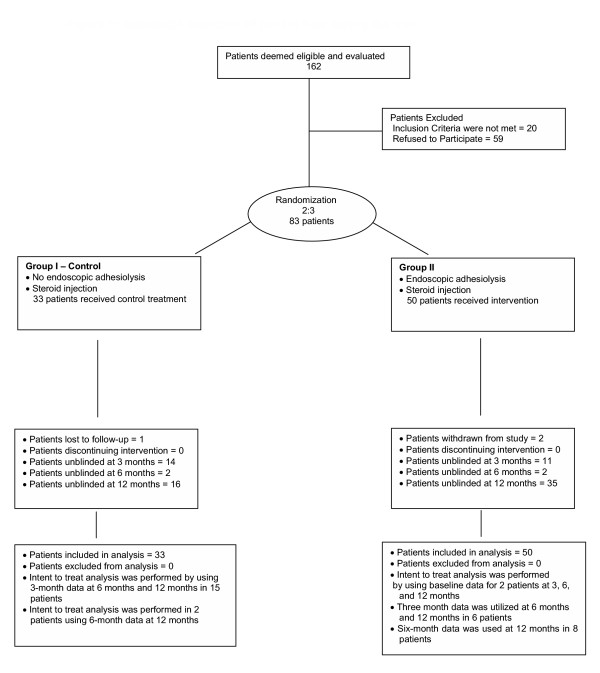
Schematic depiction of patient flow during the trial.

A diagram illustrating flow of the trial is depicted as Figure 1. In the control group, one patient was lost to follow-up after 3 months. In the intervention group, two patients withdrew from the study. One patient experienced no improvement, withdrew from the study, and underwent further surgical intervention. The second patient in the intervention group reported no significant relief and withdrew from the study and refused further follow-up. Intent to treat analysis was performed by using baseline or last follow-up data in both groups. All patients received only one treatment during the study period. Patients were considered withdrawn if they received any other interventional techniques. Last follow-up was utilized for analysis with 3-month data at 6 months and 12 months in 15 patients, and 6-month data at 12 months in two patients in the control group. In the intervention group, baseline data was utilized in two patients. In the intervention group, of the 11 patients unblinded at 3 months, five of them participated in outcomes assessment at 6 months and 12 months while six of them participated only at 6 months assessment. Thus, 3 month assessment results were utilized at 6 months and 12 months, whereas the results of 6 month assessments were utilized at 12 months in eight patients (six patients from 3 month unblinding and two patients from 6 month unblinding).

### Demographic characteristics

Table 1 illustrates the demographic characteristics. All the patients presented with back and lower extremity pain. Most patients had pain with unilateral symptoms, while bilateral symptoms were seen in 12% of the patients in each group.

**Table 1:** Demographic characteristics

		**Group I**	**Group II**
Number of patients		33	50
Age (Years)	Mean ± SD	47 ± 9.4	50 ± 9.0
Gender	Male	54% (18)	36% (18)
	Female	46% (15)	64% (32)
Height (Inches)	Mean ± SD	66 ± 3.6	66 ± 3.5
Weight (Lbs)	Mean ± SD	181 ± 42.4	174 ± 36.8
Duration of pain (years)	Mean ± SD	12.4 ± 5.9	11.8 ± 6.5
Mode of onset of the pain	Traumatic	39% (13)	46% (23)
	Non-traumatic	61% (20)	54% (27)
Back and lower extremity pain		100% (33)	100% (50)
Bilateral pain		12% (4)	12% (6)
History of previous surgery		73% (24)	84% (42)
Epidural fibrosis on MRI		73% (24)	84% (42)
Disc herniation on MRI		12% (4)	10% (5)

### Procedural characteristics

In the control group, of the four patients (12%) with bilateral back and lower extremity pain, two patients received 12 mg of betamethasone and two patients received 80 mg of methylprednisolone. Of the remaining 29 patients, 16 patients received 6 mg of betamethasone and 13 patients received 40 mg of methylprednisolone. The volume of contrast was 8.6 ± 1.25 mL with a range of 8 to 12 mL (Table 2).

**Table 2:** Description of procedural characteristics

		**Group I 33**	**Group II 50**
Betamethasone			

12 mg		6% (2)	4% (2)
6 mg		48% (16)	56% (28)
Total		55% (18)	60% (30)
Methylprednisolone			
80 mg		6% (2)	8% (4)
40 mg		39% (13)	32% (16)
Total		45% (15)	40% (20)
Contrast in mL	Mean ± SD	8.6 ± 1.25	11.2 ± 2.74
	Range	8 – 12	8 – 16
Sodium chloride solution for irrigation in mL	Mean ± SD	None	55.0 ± 11.07
	Range		35 – 70

() indicates number of patients

In the intervention group, adhesiolysis was performed bilaterally in six patients (12%). Adhesiolysis was performed at one level in two patients, at two levels in 47 patients, and at four levels in one patient. Unilateral adhesiolysis was performed in only one patient at L4 level. No bilateral adhesiolysis was performed at L4. Most commonly, adhesiolysis was performed at L5 and S1. The volume of sodium chloride solution injected was 55.0 ± 11.07 mL with a range of 35 to 70 mL. The volume of contrast was 11.2 ± 2.74 mL with volumes ranging from 8 to 16 mL. There were no cases of subarachnoid blockade identified prior to injection of local anesthetic and steroid. Thus, although it was available as part of the protocol, a Racz catheter was not used for any patient procedure in the study. Twelve milligrams of betamethasone in 8 mL of 1% lidocaine was injected in two patients and 80 mg of methylprednisolone in 8 mL of 1% lidocaine was injected in four patients, 6 mg of betamethasone in 4 mL of 1% lidocaine was injected in 28 patients, and 40 mg of methylprednisolone in 4 mL of 1% lidocaine was injected in 16 patients (Table 2).

### Outcome measures

**Figure 2:**
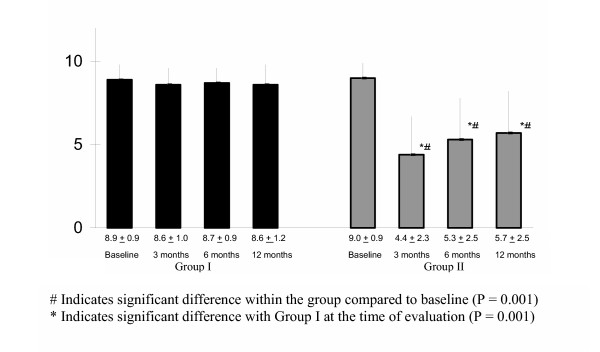
Outcome measurement based on Visual Analogue Scale report.

A significant proportion of patients in the spinal endoscopic adhesiolysis group showed pain relief compared to the control group, as well as compared to the baseline findings (Fig 2).

Significant pain relief (≥ 50%) in months was calculated for both groups. Calculations for all patients showed that significant relief was seen for 0.7 ± 0.73 months in the control group, whereas, 7.6 ± 4.7 months of relief was noted for the intervention group. Significant pain relief was longer in the intervention group. Duration of significant relief (≥ 50%) (mean ± SD) was 9.3 ± 3.6 months in patients considered as successful (40 of 50).

**Figure 3:**
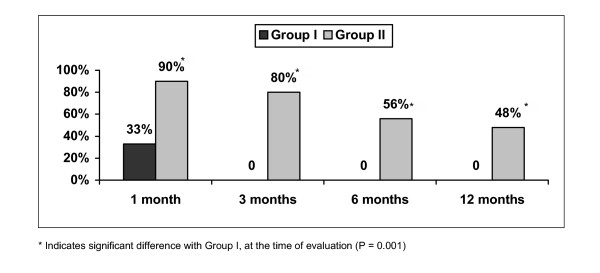
Proportion of patients with significant relief (≥ 50%) at 1 month, 3 months, 6 months and 12 months.

In the control group, the proportion of patients with significant relief greater than 50% at 1 month was 33%, and at 3 months, 6 months, and 12 months was 0. By contrast, in the intervention group relief was 90% at 1 month, 80% at 3 months, 56% at 6 months, and 48% at 12 months (Fig. 3).

**Figure 4:**
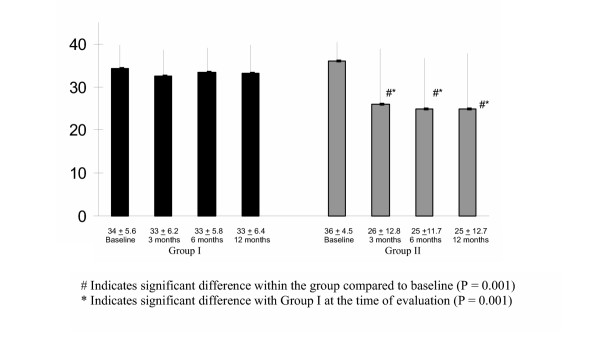
The Outcome Measurement Based on Oswestry Disability Index.

Functional outcome measurement was carried out based on Oswestry Disability Index 2.0. Significant improvements were seen in the intervention group compared to baseline in the same group, as well as compared to the control group (Fig. 4).

Analysis of range of motion evaluations showed significant improvements in the intervention group compared to the baseline, as well as the control group at intervals of 3 months, 6 months, and 12 months (Table 3).

**Table 3:** Analysis of range of motion evaluation

		**Baseline**		**3 months**		**6 months**		**12 months**	
		
		Group I	Group II	Group I	Group II	Group I	Group II	Group I	Group II
		
		33	50	33	50	33	50	33	50
Flexion (Normal 60°)	Mean ± SD	25.4 ± 10.0	25.9 ± 11.4	26.6 ± 10.3	35.8*^# ^± 11.7	25.8 ± 10.4	36.7*^# ^± 13.7	25.6 ± 10.3	35.7*^# ^± 14.4
Extension (Normal 25°)	Mean ± SD	9.7 ± 3.9	9.0 ± 3.3	10.9 ± 5.1	14.7*^# ^± 5.3	10.5 ± 5.1	15.8*^# ^± 6.5	10.9 ± 5.3	16.3*^# ^± 7.0
Lateral Flexion (Normal 25°)	Mean ± SD	8.1 ± 2.9	8.4 ± 2.8	7.9 ± 3.0	14.0*^# ^± 5.3	7.8 ± 3.0	14.6*^# ^± 6.1	7.7 ± 2.8	15.1*^# ^± 6.8

* Indicates significant difference with Group I (P = 0.002)

^# ^Indicates significant difference within the Group compared to baseline (P = 0.001)

Table 4 illustrates psychological outcomes of depression, anxiety, and somatization derived from P-3 scores. Significant improvement was noted in psychological parameters in the intervention group compared to the control group, as well as to baseline status in the treatment group.

**Table 4:** Analysis of psychological status

		**Baseline**		**12 months**	
		Group I	Group II	Group I	Group II
		
		33	50	33	50

Depression	Diagnosis (≥ 55)	61% (20)	68% (34)	58% (19)	34%*(17)
	Score Mean ± SD	56.9 ± 8.8	57.0 ± 9.9	55.5 ± 10.6	47.8*^# ^± 10.4

Anxiety	Diagnosis (≥ 56)	58% (19)	62% (31)	55% (18)	28%*(14)
	Score Mean ± SD	55.6 ± 10.6	55.9 ± 11.9	54.9 ± 9.9	46.8*^# ^± 12.1

Somatization	Diagnosis (≥ 56)	58% (19)	74% (34)	52% (17)	30% (18)
	Score Mean ± SD	55.4 ± 8.9	56.6 ± 11.4	55.9 ± 10.4	47.8*^# ^± 12.3

* Indicates significant difference with Group I (*P *= < 0.05)

# Indicates significant difference with Baseline values within the Group (*P *= < 0.001)

Patients were evaluated for opioid intake, which was rated from none to significant as described in the methods section. Significant opioid intake was 40% in Group II at the end of 12 months, compared to 74% at baseline. For Group I, significant opioid usage was 55% at 12 months, compared to 61% at baseline.

Evaluation of employment status showed that in the intervention group employment increased to 32% at 12 months from 2% at baseline as compared to 6% at baseline and at 12 months in the control group. As illustrated in Table 5, almost all the patients deemed employable in the intervention group were employed at 12 months, in contrast to no change noted in the control group. In addition, in the intervention group, eight patients disabled at baseline were also employed at 12 months. There were no patients in this study with active workers' compensation injury cases or litigation.

**Table 5:** Change in proportion of patients with employment status

Employment Status	Group I		Group II	
	Baseline	At 12 months	Baseline	At 12 months

Employed	2 (6%)	2 (6%)	1 (2%)	16 (32%)*
Unemployed	2 (6%)	2 (6%)	8 (16%)	1 (2%)
Housewife	2 (6%)	2 (6%)	1 (2%)	1 (2%)
Disabled	26 (79%)	26 (79%)	38 (76%)	30 (60%)
Over 65 (yrs)	1 (3%)	1 (3%)	2 (4%)	2 (4%)
Total	33	50	33	50

*Indicates significant difference (*P *< 0.01)

### Blinding

The blinding was judged to be satisfactory. Following the treatment, and within one hour prior to discharge, patients were asked what treatment they believed they had received. Twenty-six of 33 patients in Group I and 42 of 50 patients in Group II believed they had received spinal endoscopy. Two patients in Group I and two patients in Group II were unable to theorize as to which procedure they may have received. The remaining patients guessed the wrong treatment. There was no significant difference among the groups as to whether they believed they had received the endoscopy or epidural steroid injection.

### Adverse events

There was one case of subarachnoid block in Group II, identified after completion of the procedure and injection of local anesthetic and steroid. No adverse effects were noted in this patient. There were no other adverse events noted.

## Discussion

This randomized, double-blind, controlled evaluation demonstrated that following spinal endoscopic adhesiolysis a significant proportion of patients with chronic, refractory low back and lower extremity pain experienced significant pain relief (≥ 50%) at 3 months (80%), 6 months (56%), and at 12 months (48%), compared to the control group with only 33% of patients showing improvement at 1 month, and none thereafter. Associated improvements in VAS scores, Oswestry Disability Index, range of motion, and psychological status were also noted as compared to baseline measurements and results of the control group. These results are important in that the patients in this study represented a subset of patients who have not only failed multiple conservative modalities of management but also lacked significant, or long-term, response to fluoroscopically-directed epidural steroid injections and one-day percutaneous adhesiolysis.

Numerous studies have evaluated the effectiveness of spinal endoscopic adhesiolysis [24-29,31]; however, these studies utilized heterogenous inclusion criteria. Igarashi et al [29] evaluated patients with degenerative lumbar spinal stenosis. Manchikanti et al [26] evaluated only patients with a history of previous surgical intervention. In another study, Manchikanti et al [27] included patients who had not previously undergone surgery (a total of 16%). Dashfield et al [31] included only non-surgical patients. Geurts et al [24] reported results of spinal endoscopic adhesiolysis in 20 patients suffering with chronic low back pain. They reported > 50% reduction in pain in 40% of the patients at 3 months, and 35% at 6, 9, and 12 months. Richardson et al [25] reported results in 38 patients, with 19 of those patients identified with failed back surgery syndrome. They reported significant improvement based on Visual Analogue Scale and functional abilities. However, they have not reported data with regards to the proportion of patients with sustained relief at various time periods. Manchikanti et al [26,27] in two different studies, reported 75% relief at 3 months, 40% at 6 months, and 22% at 12 months in post-lumbar laminectomy patients; and, in a heterogenous group of patients including both post laminectomy and non-surgical patients, 52% of the patients at 3 months, 21% of the patients at 6 months, and 7% of the patients after 12 months. Igarashi et al [29] evaluated 58 patients with degenerative lumbar spinal stenosis, dividing them into two groups based on the presenting symptoms of either a monosegmental group (n = 34) or a multisegmental group (n = 24). They showed that relief of low back pain was observed up to 12 months after epiduroscopy in both groups, whereas relief of leg pain was evident up to 12 months after epiduroscopy in the monosegmental group, and up to 3 months after epiduroscopy in the multisegmental group. Dashfield et al [31] compared caudal epidural steroid injections with targeted steroid placement during spinal endoscopy for chronic sciatica in a prospective, randomized, double-blind trial. They randomized 60 patients with a 6-to 18-month history of sciatica to either a targeted epidural local anesthetic and steroid placement with a spinal endoscope, or caudal epidural local anesthetic and steroid placement. They defined sciatica as pain in the distribution of lumbar nerve root, accompanied by neurosensory and motor deficits, with or without back pain. They excluded patients with history of previous spinal surgery, coagulopathy, progressive motor neuron disorders or peripheral vascular disease, and patients receiving epidural corticosteroid injections within 3 months. No significant differences were found between the groups for any of the measures at any time. However, there were significant differences within both groups compared with pre-treatment values. The results of the present evaluation may not be compared to either the studies by Igarashi et al [29] or by Dashfield et al [31].

Igarashi et al [29] evaluated patients only with spinal stenosis. One-day percutaneous adhesiolysis also was shown to be effective in refractory spinal stenosis [44]. However, Igarashi et al [29] did not treat their patients with spinal stenosis with percutaneous adhesiolysis, which is considered as a safer and more effective procedure. The study by Dashfield et al [31] was performed in patients who were not expected to have epidural fibrosis and who had not been treated with either epidural steroid injections or with 1-day or 3-day percutaneous adhesiolysis. Consequently, there was no significant difference noted between caudal epidural steroid injections and targeted steroid placement with spinal endoscopy. In clinical practice in the United States, invasive intervention with spinal endoscopy as an initial treatment is not widely accepted.

The most common and worrisome complications of spinal endoscopy with adhesiolysis and injection of corticosteroids are related to dural puncture, spinal cord compression, intravascular injection, vascular injury, cerebral vascular or pulmonary embolus, infection, steroids, instrumentation with endoscope, and administration of high volumes of fluids potentially resulting in excessive epidural hydrostatic pressures, resulting in blindness, neurapraxia, numbness, intravascular injections, brain damage, and death [23-30,45-58]. Even though no major complications have been noted in this study, it is recommended that all precautions be undertaken, along with exhaustion of other modalities of treatments prior to embarking on spinal endoscopic adhesiolysis considering the safety and cost. The cost of the endoscope and the procedure are higher than either caudal epidural steroid injections or 1-day or 3-day percutaneous adhesiolysis procedure. The safety and effectiveness of 1-day and 3-day percutaneous adhesiolysis has been demonstrated [21,22].

The present evaluation utilized early unblinding in some patients, did not include a placebo group, and adapted a randomization ratio of 2:3 instead of 1:1. Considering the difficulties of recruiting patients to a double-blind trial, the authors considered the best way to recruit patients and give them a reasonable level of comfort was to offer additional treatment(s) if they failed the study, rather than allowing them to suffer for a year. Based on this allocation, the authors managed to include an acceptable number of patients. The control group for the study was not a true placebo group since interventions of caudal epidural steroid injections were used; nevertheless, the injections were ineffective in these patients. One of the objectives of the study was to demonstrate whether epidural steroid injections administered after adhesiolysis are effective as opposed to traditional or fluoroscopically-directed epidural steroid injections. In addition, this also served to provide a level of comfort to patients enrolled in the study since they knew they would receive some type of active treatment rather than a placebo. The authors believed that this type of randomization with a control group receiving standard treatment to be more effective and provide optimal results. A randomization process with a 2:3 ratio was selected to convince patients to enroll in the study, as they would have a higher chance of being included in a treatment group rather than a control group. The statistical validity was maintained throughout the study and an intent-to-treat analysis was incorporated in the study.

Trials of healthcare interventions are often described as either explanatory or pragmatic [40,41]. Explanatory trials generally measure efficacy – the benefit a treatment produces under ideal conditions. Consequently, explanatory trials often use carefully-defined subjects in a well-controlled research setting. By contrast, pragmatic trials, also known as practical clinical trials, measure effectiveness – benefit the treatment produces in routine clinical practice. Tunis et al [40] commented on the prevalence and significance of gaps in knowledge about clinical effectiveness of interventions and suboptimal evidence available to answer the critical questions. Most systematic reviews performed in interventional pain management include studies providing data not applicable to patients treated in typical practice settings. Consequently, limited quantity and quality of available scientific information impedes the efforts of public and private health insurers in developing evidence-based coverage policies for many new and existing technologies [59,60].

The substantial differences between explanatory and pragmatic trials illustrate a paradigm shift to clinical practice. Patient selection in an explanatory approach is based on the principles of homogenous population, primarily aiming to further scientific knowledge. However, in a pragmatic or practical clinical trial, the design reflects variations between patients that occur in real life clinical settings, and aims to inform choices between treatments. The authors consider this trial to be close to pragmatic or practical rather than explanatory. Even with appropriate randomization, the major focus of clinical research in the modern era of medicine, multiple other sources of bias may affect results. In this study, independent assessment was utilized. However, without a placebo treatment, in pragmatic approaches, the treatment response is the total difference between two treatments, including both treatment and associated placebo effects, as this will best reflect the likely clinical response in practice [22,30,58,61-65]. Practical clinical trials are expected to best address questions about the risks, benefits, and cost of an intervention as they would occur in routine clinical practice [41]. Thus, the most distinctive features of practical clinical trials are that they select patients from practices, either simulating actual practices or actual clinical practices. In addition, practical clinical trials often are designed to compare viable alternative clinical strategies. This study achieves both the distinctive features of practical clinical trials by selecting the population from an actual clinical practice and also by comparing viable alternative clinical strategies.

This procedure may be considered as a replacement for large bore catheter for percutaneous adhesiolysis, as we have not derived any diagnostic information. However, visualization of the scar tissue and freeing of the scar tissue from the nerve root may provide some additional benefit. The present day available catheters for percutaneous adhesiolysis are smaller bore. Consequently, spinal endoscopy with larger bore and improved flexibility appears to have a role. Percutaneous adhesiolysis has been shown to be an effective and safe procedure [21,22,45,46]. However this study went beyond percutaneous adhesiolysis and selected the patients after insufficient response after 1-day percutaneous adhesiolysis.

The issue remains for the patients who have had successful relief for 6 months or 12 months with regards to further treatment when pain returns and functional status deteriorates. Based on the present literature, with proper indications and precautions, the procedure may be repeated after approximately 6 months. In addition, the authors believe that even if patients have not responded previously to these procedures, if they have responded initially to spinal endoscopy, they may respond to 1-day or 3-day percutaneous adhesiolysis or even caudal epidural steroid injections. However, published data is not available at present to support this assumption and controlled trials are recommended to evaluate this postulate. Additional effect from spinal endoscopic adhesiolysis may be dependent on hydrostatic pressures created by the administration of sodium chloride solution which is not the case with lesser volume percutaneous adhesiolysis. However, percutaneous adhesiolysis also may be modified to accommodate this feature.

## Conclusion

This controlled trial demonstrates that spinal endoscopic adhesiolysis reduces pain and improves functional and psychological status without adverse effects up to 12 months.

## Competing interests

The author(s) declare that they have no competing interests.

## Authors' contributions

LM conceived and designed the study, processed the data and wrote the manuscript.

MVB participated in the study's design and revised the manuscript.

JJR participated in its design and collected the clinical data.

VSP performed the statistical analysis.

KSD collected the clinical data.

CDM collected the clinical data.

DEB participated in the procedure.

SRW participated in the procedure.

All authors read and approved the final manuscript.

## Pre-publication history

The pre-publication history for this paper can be accessed here:


